# FAS-ligand regulates differential activation-induced cell death of human T-helper 1 and 17 cells in healthy donors and multiple sclerosis patients

**DOI:** 10.1038/cddis.2015.100

**Published:** 2015-05-07

**Authors:** M T Cencioni, S Santini, G Ruocco, G Borsellino, M De Bardi, M G Grasso, S Ruggieri, C Gasperini, D Centonze, D Barilá, L Battistini, E Volpe

**Affiliations:** 1 Neuroimmunology Unit, Santa Lucia Foundation, Rome, Italy; 2 Cell Signaling Unit, Santa Lucia Foundation, Rome, Italy; 3 Multiple Sclerosis Centre, Santa Lucia Foundation, Rome, Italy; 4 Department of Biology, University Tor Vergata, Rome, Italy; 5 Department of Neuroscience, University Tor Vergata, Rome, Italy; 6 Department of Neuroscience "Lancisi", San Camillo Hospital, Rome, Italy; 7 Neuroimmunology and Synaptic Plasticity Unit, Santa Lucia Foundation, Rome, Italy

## Abstract

Functionally distinct T-helper (Th) subsets orchestrate immune responses. Maintenance of homeostasis through the tight control of inflammatory Th cells is crucial to avoid autoimmune inflammation. Activation-Induced Cell Death (AICD) regulates homeostasis of T cells, and it has never been investigated in human Th cells. We generated stable clones of inflammatory Th subsets involved in autoimmune diseases, such as Th1, Th17 and Th1/17 cells, from healthy donors (HD) and multiple sclerosis (MS) patients and we measured AICD. We find that human Th1 cells are sensitive, whereas Th17 and Th1/17 are resistant, to AICD. In particular, Th1 cells express high level of FAS-ligand (FASL), which interacts with FAS and leads to caspases' cleavage and ultimately to cell death. In contrast, low FASL expression in Th17 and Th1/17 cells blunts caspase 8 activation and thus reduces cell death. Interestingly, Th cells obtained from healthy individuals and MS patients behave similarly, suggesting that this mechanism could explain the persistence of inflammatory IL-17-producing cells in autoimmune diseases, such as MS, where their generation is particularly substantial.

T-helper (Th) cells are responsible for the orchestration of the adaptive immune response. In particular, Th1 cells, which produce interferon (IFN)-*γ*, mediate responses against infection by intracellular microbes; Th2 cells, which secrete interleukin (IL)-4, IL-5 and IL-13, activate the response for clearance of helminthes; Th17 cells, producers of IL-17A and IL-17F, protect mucosa from any bacterial or fungal infection.^1^ However, persistent or uncontrolled Th cell responses are often associated with pathological states and tissue damage. An excessive Th2 cell response is implicated in atopic diseases, such as asthma,^2^ whereas abnormal Th1 cell responses are involved in chronic inflammation and mediate several autoimmune diseases.^3, 4^ In the past years, a major role in autoimmune pathology has been assigned to Th17 cells.^5, 6, 7, 8^ The concept that Th17 cells are responsible for driving autoimmune inflammation was established when experimental autoimmune encephalomyelitis (EAE), the murine model of MS, was induced by passive transfer of IL-17- producing memory-activated T cells.^7^ It has been suggested that Th17 cells are critical in sustaining tissue damage, whereas Th1 cells may be important in the initiation of the inflammatory response by inducing a state of increased vascular adherence that facilitates access of Th17 cells to the central nervous system (CNS).^8^ In this context, cells producing both IL-17 and IFN-*γ*, called Th1/17, are emerging as potentially relevant in the pathogenesis of MS.^9^ The persistence of effector Th cells in pathological states may be related to alterations of their generation or of their survival processes. The factors that regulate the generation of human Th1 and Th17 cells are well known,^10, 11, 12^ whereas the origin of Th1/17 cells is less clear. There are some evidence indicating that the Th1/17 profile originates from Th17 cells cultured in the presence of IL-12.^13^ However, limited information is available on the homeostatic signals that regulate their survival and limit their expansion. Cell death is an important mechanism for the maintenance of immune homeostasis. In fact, during a physiological immune response, programmed cell death (apoptosis) has the important role of deleting potentially pathogenic autoreactive lymphocytes from the circulation and tissues, limiting the tissue damage inevitably caused by immune responses.^14^ Moreover, T-cell receptor (TCR) re-stimulation of previously activated and expanded T cells in the absence of appropriate co-stimulation induces activation-induced cell death (AICD).^15, 16^ AICD involves stimulation through CD95 (Fas), TNFR1, TRAILR and other mechanisms independent of cell death receptors.^17^ Induction of apoptosis following re-stimulation through the TCR is also important for removal of overly activated T cells, such as autoreactive T cells in autoimmune diseases. AICD has been investigated in murine Th cells, revealing that Th17 cells are more resistant than Th1 cells. The resistance of Th17 cells to AICD has been associated with lower expression of FASL and with overexpression of the anti-apoptotic protein caspase-8 (FLICE)-like inhibitory protein (FLIP) by Th17 cells.^18, 19^ Similar results were obtained comparing sensitivity of murine Th1 and Th17 cells with FAS-mediated apoptosis^20^ and with antigenic re-stimulation,^18^ and it has been shown that FASL, TRAIL and TIM3 are expressed at levels significantly higher in Th1 than in Th17 cells.^21^ However, AICD in human Th cells has never been investigated. In this study, we have analysed cell death receptor pathways in human Th profiles known to be involved in autoimmune disorders, such as Th1, Th17 and Th1/17 cells.^22^ We found that transcription of FASL is a crucial step for the regulation of Th cell death sensitivity. Importantly, we observed that Th1/17 cells express low levels of FASL and show resistance to AICD, which renders them more similar to Th17 than to Th1 cells.

## Results

### Human Th1 cells are more sensitive to TCR-mediated cell death than other Th profiles

In order to study cell death induced by TCR stimulation in human Th clones, we sorted by flow cytometry single memory T cells from healthy donors (HD) (Supplementary Figure S1A), and we selected growing cell clones based on the stable and high expression of specific cytokines (Supplementary Figures S1B and C): Th1 as IFN-*γ*-producing cells; Th17 as IL-17-producing cells; Th1/17 as IFN-*γ* and IL-17-producing cells; and Th0 as non-producers of either IFN-*γ* or IL-17 (Supplementary Figures S1A and B).

**Figure 1:**
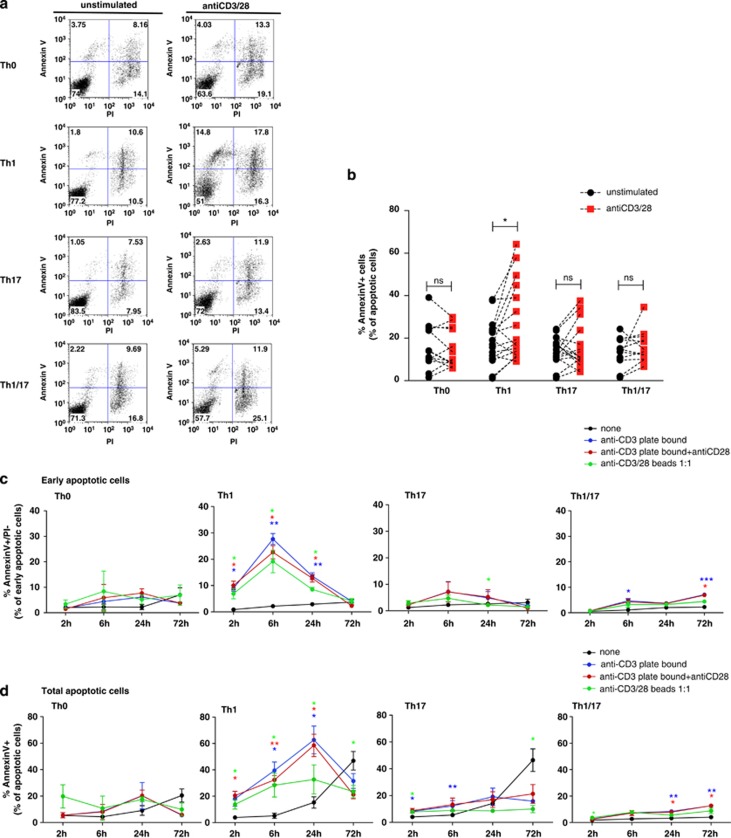
Th1 cells are more sensitive to TCR-mediated cell death than other Th profiles. Pools of Th0, Th1, Th17 and Th1/17 clones from the same donor were stimulated with anti-CD3-28 beads, anti-CD3 plate bound and soluble anti-CD28. At 24 h (**a** and **b**) and 2-6-24-72 h (**c** and **d**) after stimulation, clones were stained for Annexin V and PI and then analysed by flow cytometry. The percentage of Annexin V^+^/Propidium Iodide (PI)^−^ cells (early apoptotic cells) (**c**) and Annexin V^+^ cells (total apoptotic cells) (**b** and **d**) is reported in the cumulative graph. A representative experiment (**a**) and cumulative data of 16 (**b**) or three (**c** and **d**) independent experiments performed on 16 (**b**) or three (**c** and **d**) healthy donors are presented. A paired *t*-test was used to compare sample condition (defined by different colours) with unstimulated (black). *P<0.05; **P<0.005

Clones were activated with anti-CD3 and anti CD28, and apoptosis was measured by flow cytometry. We found that human Th17 and Th1/17 cells are similarly resistant to AICD and that Th1 cells are the most sensitive Th cells to AICD (Figures 1a and b).

Although other forms of cell death induced by TCR have been described,^23^ classical AICD is mediated by the stimulation with anti-CD3 in the absence of co-stimulatory signals.^17^ Thus, we compared in our model different types of TCR stimulation: plate bound anti-CD3, plate bound anti-CD3 with soluble anti-CD28 and anti-CD3/28-coated beads. Following stimulation of cells for different time points, we measured the percentage of Annexin V^+^/propidium iodide (PI)^−^ cells (early apoptotic cells) (Figure 1c) and Annexin V^+^ cells (total apoptotic cells) (Figure 1d) by flow cytometry. We confirmed that human Th1 clones are particularly sensitive to apoptosis: plate-bound anti-CD3 induces 8.9±1.1 percent of early apoptotic cells at 2 h, reaching a peak of 27.6±2.0 percent at 6 h after stimulation (Figure 1c). The increase in total apoptotic cells is clear at 6 h (39.4±6.4 percent) and 24 h after stimulation (62.7±10.5% percent) (Figure 1d). The presence of anti-CD28 does not protect Th1 cells from anti-CD3-mediated apoptosis (Figures 1c and d). In contrast, apoptosis is not significantly induced with either anti-CD3 alone or anti-CD3-28 in Th0, Th17 and Th1/17 cells (Figures 1c and d).

Thus, human Th1 and Th17 subsets differ in their sensitivity to cell death mediated by TCR stimulation. In order to address whether the differential sensitivity to TCR-mediated cell death was related to dissimilarities in TCR-mediated activation, we evaluated the expression of activation markers, such as CD25 and CD69, in the same experimental conditions. We found that Th1 and Th17 cells are similarly activated after 24h of stimulation (Supplementary Figure S2). Moreover, we found that, although the fraction of proliferating cells is similar in all different Th clones, the proliferation index of Th17 cells is lower compared with that of Th0 and Th1 clones after TCR stimulation (Supplementary Figure S3). Thus, the low proliferating rate of Th17 clones could contribute to their resistance to activation of the cell death machinery. Finally, as in recent years CD161 expression has been used to discriminate Th1 cells between classical and non-classical Th1 cells,^24^ we analysed whether cell death sensitivity was similar in these two populations, and we found that both subtypes of Th1 are more sensitive to AICD than are Th17 cells (Supplementary Figure S4A and B).

### The FAS–FASL pathway is involved in AICD of human Th1 cells

**Figure 2:**
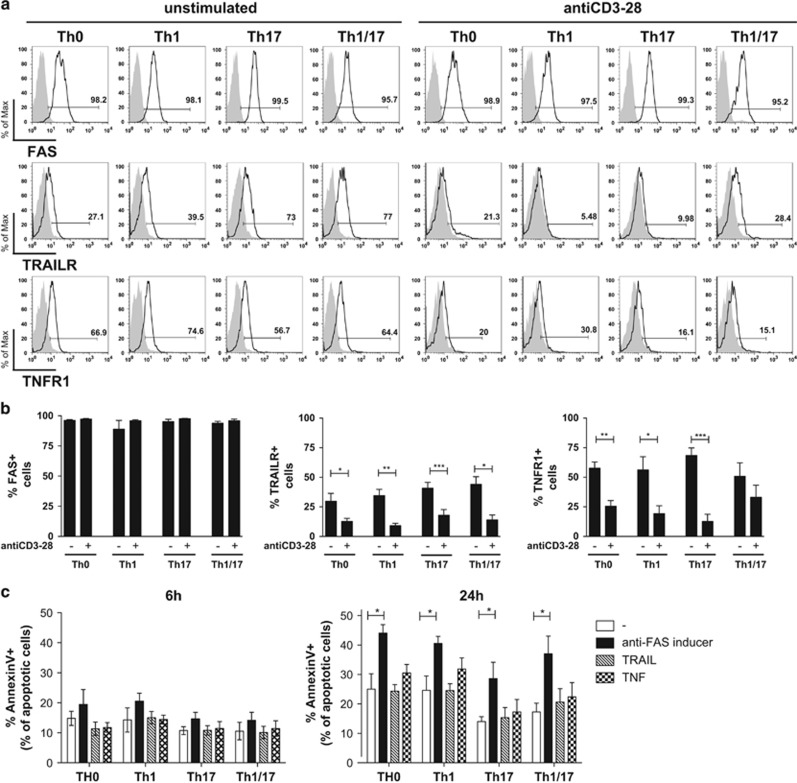
Cell-death receptor expression and functions are similar in all Th profiles. Pools of Th0, Th1, Th17 and Th1/17 clones were stimulated with anti-CD3-28 (**a** and **b**) or anti-FAS inducer 0.5 *μ*g/ml, iz-TRAIL 10 *μ*g/ml, TNF-*α* 1 *μ*g/ml and after (**c**). After 24 h of stimulation, the expression of FAS, TRAILR and TNFR1 was analysed by flow cytometry (**a** and **b**). After 6–24 h of stimulation, apoptosis was analysed by staining with Annexin V and PI, and flow cytometry (**c**). A representative experiment of receptor expression is shown in panel **a**; mean values (±S.D.) of five (**b**) or four independent experiments performed on five (**b**) or four (**c**) healthy donors are presented in panels **b** and **c**. A paired *t*-test was used to compare sample conditions. *P<0.05; **P<0.005; ***P<0.001

As AICD in T cells involves stimulation through cell-death receptors CD95 (FAS), TNFR1 and TRAILR,^17^ we hypothesised that a differential expression and/or function of death receptors could be responsible for the differential sensitivity to cell death of Th1 and Th17 clones. The expression of death receptors was analysed by flow cytometry on the surface of unstimulated or TCR-stimulated Th clones. The analysis revealed that the FAS receptor is widely expressed in all Th profiles, independent from the stimulation state, whereas the receptors for TRAIL and TNF were expressed at low levels in all unstimulated clones and were further reduced by stimulation with anti-CD3-28 (Figures 2a and b). In line with these results, we found that among the cell death stimuli, including anti-FAS inducer, iz-TRAIL and TNF-*α*, only the FAS agonist was able to induce cell death in all Th profiles (Figure 2c). Importantly, we did not observe differences between distinct Th clones, neither in the expression of FAS nor in the ability of FAS agonist to induce cell death. Overall, these data indicate that the expression and function of FAS and of other cell death receptors are not responsible for the differential sensitivity to AICD of Th1, Th17 and Th1/17 cells.

**Figure 3:**
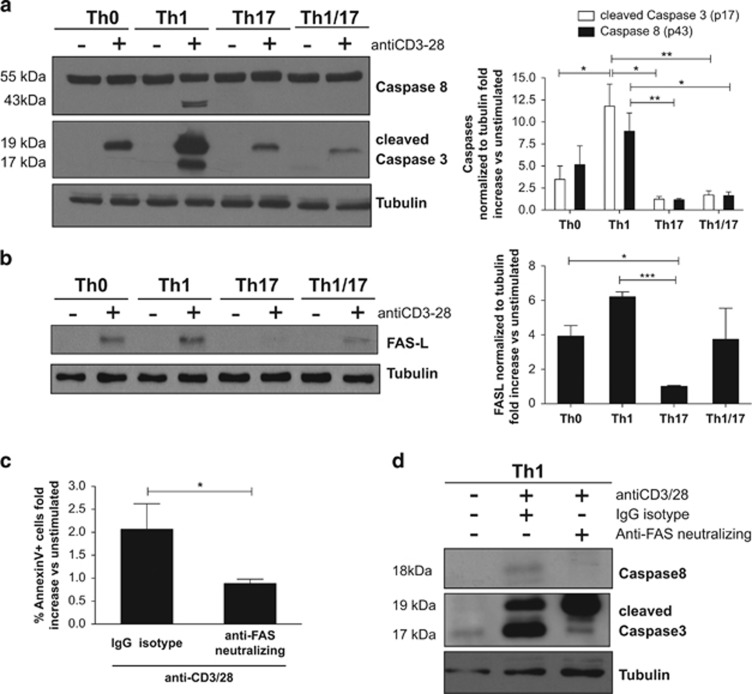
Sensitivity of Th1 cells to TCR-mediated apoptosis involves caspases and FASL expression. Pools of Th0, Th1, Th17 and Th1/17 clones from the same donor were stimulated as indicated. At 24 h (**a, c** and **d**) or 6 h (**b**) after stimulation, expression of caspases (**a** and **d**) or FASL (**b**) was analysed by western blot and densitometry; apoptosis was analysed by flow cytometry (**c**). Results are expressed as mean values (±S.D.) of eight (**a**), four (**b**) and six (**c**) of independent experiments performed on eight (**a**), four (**b**) and six (**c**) HD; representative experiment is reported (**d**). A paired *t*-test was used to compare sample conditions. *P<0.05, **P<0.005, ***P<0.001

Consistent with previous data, we found that Th1 cells are the only Th profile expressing high levels of cleaved caspase-8 (43 kDa) after anti-CD3/28 stimulation (Figure 3a), indicating an involvement of caspase-8 in the apoptotic pathway of Th1 cells. Caspase-8 (43 kDa) derives from procaspase-8 (55 kDa), which is composed by DED domain, p18 and p10 subunits. The activation process requires steps of dimerisation and autocleavage, leading to the release of the p18 and p10 subunits that will be assembled in the fully active caspase-8 tetramer.^25^ The autoprocessing events lead to the ordered production of the p10 (small) and p43 (DEDs-p18) subunits first and then to the release of the p18 (large) subunit. p43 release is an early event in caspase 8 activation, and it is also easily detectable, but its presence does not exactly correspond to the full activation of caspase-8. Thus, we verified that its presence was associated with caspase-8 activation by performing a fluorimetric activity assay for caspase-8, and results are reported in supplementary Supplementary Figure S5A.

Moreover, we observed high levels of cleaved caspase-3 (17 kDa) in Th1 cells stimulated with anti-CD3/28. Although the 19-kDa fragment of caspase-3 is present in all activated Th clones, the effective activation of caspase-3 requires proteolytic processing of its inactive zymogen into the activated 17-kDa fragment.^26^ Validation of caspase-3 activity by the fluorimetric assay and cleavage analysis of two known downstream effectors of caspase-3, PARP^27^ and ATM^27, 28^ (Supplementary Figures S5B and C), support the conclusion that TCR stimulation triggers caspase-8 and caspase-3 activation preferentially in Th1 compared with Th17 cells.

**Figure 4:**
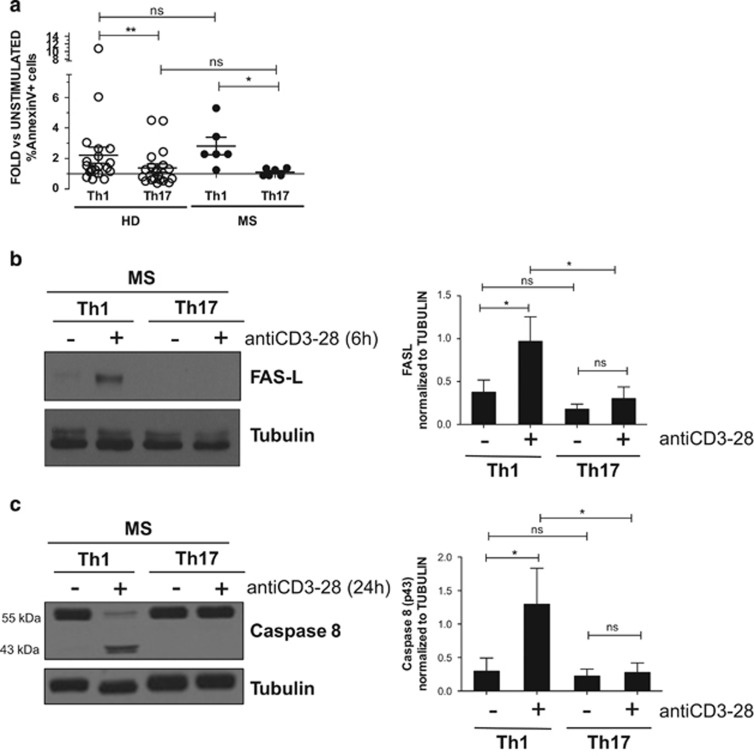
Th cells from MS patients and HD are similarly sensitive to AICD. Pools of Th1 and Th17 clones from HD and RR-MS (active) were stimulated as indicated (**a**, **b** and **c**). At 24 h after stimulation with anti-CD3/28, clones were stained for Annexin V and PI and analysed by flow cytometry. Data are expressed as fold *versus* unstimulated cells of independent experiments performed on different donors (**a**). At 6 h and 24 h after stimulation, the levels of FASL (**b**) and caspase-8 (**c**), respectively, were evaluated by western blot and analysed by densitometry in clones from MS patients. Representative and mean values (±S.D.) of four independent experiments performed on four MS patients (**b** and **c**) are reported. A paired *t*-test was used to compare sample conditions. An unpaired *t*-test was used to compare HD and MS patients. *P<0.05

Remarkably, we observed that TCR stimulation triggers the induction of the expression of FASL selectively in Th1 cells (Figure 3b). The significant difference between Th1, Th1/Th17 and Th17 clones in the expression of FASL suggested that the FAS–FASL pathway may mediate the apoptotic pathway of Th1 cells. We verified this hypothesis by using a specific antibody neutralising FAS during AICD of Th1 cells. We analysed the final cleaved products of caspase-8 (18 kDa), and we found a significant reduction in apoptosis and in caspase cleavage in stimulated Th1 cells treated with the neutralising antibody (Figures 3c and d). Given the important role that this phenomenon could have in autoimmune diseases, we performed similar experiments in Th clones generated from MS patients. We compared the percentage of dead cells in Th1 and Th17 clones derived from MS patients following stimulation. Interestingly, the differential cell death sensitivity of Th1 and Th17 cells was also observed in cells derived from MS patients after 24 h of stimulation (Figure 4a). Moreover, a strong cleavage of caspase 8 and FASL expression were significantly induced in Th1 compared with Th17 cells derived from MS patients (Figures 4b and c), indicating that similar pathways are involved in death of Th cells from HD and MS patients.

### FASL transcription is reduced in Th17 compared with Th1 cells

**Figure 5:**
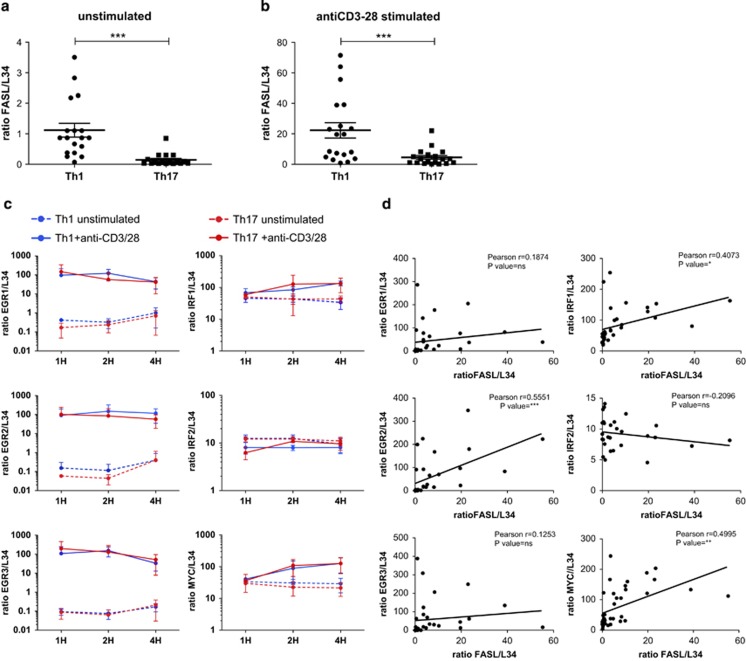
FASL transcription is reduced in Th17 compared with Th1 cells. Expression of *FASL, EGR1, EGR2, EGR3, IRF1, IRF2 and MYC* in pools of Th1 and Th17 clones was analysed by real-time PCR. Threshold cycle values were normalised to mRNA of ribosomal protein *L-34* gene. Data are mean±S.D. of independent experiments performed on independent donors (**a**–**c**). Graphs of *EGR1, EGR2, EGR3, IRF1, IRF2 and MYC* transcript levels, obtained from six independent experiments (pools of Th1 and Th17 clones unstimulated and stimulated with anti-CD3-28), were correlated to *FASL* transcript levels, using Pearson's correlation. R indicates correlation coefficient (**d**)

Given the importance of FASL in the regulation of the sensitivity to cell death of Th1 and Th17 cells, we investigated the transcriptional levels of FASL in human Th profiles. Interestingly, we found that human Th1 cells express higher basal levels of FASL transcript compared with Th17 cells (Figure 5a). Moreover, FASL transcript was increased after TCR stimulation in both profiles (Figure 5b). However, the differential expression of FASL transcript between Th1 and Th17 cells persisted, indicating that a differential regulation of FASL transcription modulates the sensitivity to cell death of Th1 and Th17 cells (Figures 5a and b). Other mechanisms known to inhibit apoptosis, such as BCLXL and FLIP expression,^28, 29^ are most likely not involved in cell death sensitivity of human Th cells. Indeed, we observed a similar induction of BCLXL after stimulation and a constant basal level of FLIP in Th1, Th17 and Th1/17 profiles (Supplementary Figure S6).

In order to investigate the potential regulating factors of FASL transcription, we analysed the expression of molecules involved in FASL induction,^30^ such as EGR1, EGR2, EGR3,^31^ IRF1, IRF2^32^ and MYC^33^ by quantitative real-time PCR. The expression of EGR1, EGR2, EGR3, IRF1 and MYC was induced after stimulation in both Th profiles, whereas IRF2 expression was not modulated by TCR stimulation (Figure 5c). Moreover, the correlation between levels of those transcripts and FASL in Th1 and Th17 cells revealed that the expression levels of EGR2, IRF1 and MYC are significantly associated with the levels of FASL transcription (Figure 5d). However, the expression of these factors was similar in all clones, suggesting that they are not responsible for the differential transcription levels of FASL in Th1 and Th17 clones (Figure 5c).

## Discussion

Previous publications demonstrated that mouse Th1 and Th17 cells differ in their susceptibility to apoptosis.^18, 19^ The present study further analyses the differential sensitivity to cell death induction of Th1 and Th17 cells in humans. We investigated apoptosis of Th cells in response to TCR activation, and we systematically examined the mechanisms potentially involved in this process. We demonstrated that not only mouse but also human Th17 cells are more resistant to AICD than are Th1 cells. These results revealed similarities between mouse and human Th cell death sensitivity important for the potential implication of this phenomenon in human diseases. Relevant to human disease, we validated the differential cell death sensitivity between Th1 and Th17 cells in human cells derived from MS patients. As the homeostatic regulation of cell expansion by cell death is similar in HD and MS patients, the persistence of Th17 cells in MS disease may be due to altered mechanisms of pathogenic Th17 cell generation in MS compared with HD. In fact, human *in vitro* studies have indicated impaired apoptotic deletion of polyclonal and myelin-specific T cells derived from MS patients' blood.^34^ As the frequency of IL-17-producing cells is higher in MS compared with that in HD,^35^ we can hypothesise that the impaired apoptotic deletion observed in MS could be related to the higher frequency of cell death resistant compared with sensitive Th subsets in MS.

In this study, we analysed for the first time the sensitivity to AICD of Th1/17 cells. As it has been described that Th1/17 (co-producing IL-17 and IFN-*γ*) cells may be generated by the plastic Th17 cell type,^36^ we generated clones from single sorted cells, and, we selected pure clones that stably maintained their phenotype over time (Supplementary Figure S1C), excluding all unstable profiles discriminating effectively between Th subsets. In this context, we compared AICD and caspase activation in Th1, Th17 and Th1/17 cells, and we found similar resistance to AICD of Th1/17 and Th17 cells. This result is consistent with other previously described similarities between these two subtypes, such as IL-23R, CCR6, and ROR*γ*t expression, ability to provide B cell help, low cytotoxic potential and reduced susceptibility to suppression by autologous Treg cells.^13^ Moreover, our results reveal that apoptosis induced by anti-CD3 and anti-CD3-28 was similar, thus excluding a CD28-mediated AICD protection in Th17 cells, which are known to express higher levels of CD28 compared with Th1 cells.^37^

Our systematic analysis of the potential cell-death receptor mechanisms reveals an exclusive role of the FAS–FASL pathway in TCR-mediated cell death of human Th1 cells. In particular, differential FASL expression between Th1 and Th17 cells is the major mechanism regulating their differential cell death sensitivity. The similar sensitivity to FAS-mediated apoptosis of Th1, Th17 and Th1/17 cells and the exclusive expression of FASL by Th1 cells suggest that interactions between different Th cell types at the inflammatory site may induce cell death in Th17 and Th1/17 following ligation of FASL expressed by Th1 cells. In instances where generation of IL-17-producing cells is favoured or increased, accumulation of these cells in inflammatory niches may preclude interactions with FASL-expressing Th1 cells, determining an increase in cell survival and a escape from homeostatic containment. Thus, differential FASL expression may regulate the fratricide of IL-17-producing cells.

In MS, the involvement of FASL has been largely investigated, but contrasting results have been reported, and it is not clear whether the levels of FASL expression in lymphocytes from MS patients are increased or decreased compared with HD.^38, 39^ Thus, the differences in frequency of Th subsets reported in previous studies^38, 39^ may explain the discordant results on FASL expression levels in total lymphocytes from healthy donors and MS patients. In murine cells, the higher upregulation of FLIP in Th17 compared with Th1 cells regulates FASL expression and cell death sensitivity.^19, 20^ However, in human cells, we found similar levels of FLIP expression in both Th cell types, indicating that FLIP is not involved in the regulation of FASL in human Th cells. The discrepancies between human and mouse studies might also be due to the different modality of Th cell generation: we performed all the experiments on clones of memory cells amplified *in vitro* in the absence of polarising cytokines, whereas previous studies used memory cells cultured in the presence of IL-12 (Th1 cells), IL-6 and TGF-*β* (Th17 cells). FLIP expression can be modulated by cytokines in Th cells;^40^ the addition of polarising cytokines in T-cell culture could potentially modulate FLIP expression and explain the discrepancies between our results and previous mouse studies.

The correlation between FASL and EGR2, IRF1 and MYC transcript expression indicates that these factors are presumably involved in FASL transcription by Th1 cells. However, our analysis of the potential transcription factors regulating FASL transcription did not permit to identify a key factor differentially expressed in Th1 and Th17 clones, likely due to the involvement of these factors in the regulation of multiple transcripts.

Interestingly, the basal levels of FASL transcript are higher in Th1 compared with Th17 cells, indicating that a constitutive activation of this transcript makes these cells more permissive to cell death. Thus, epigenetic mechanisms could be involved in this process, finally explaining the differential transcription of FASL in Th1 and Th17 cells.

Given the central role of this phenomenon in the regulation of Th cell death, further investigations on mechanisms regulating FASL transcription in Th cells will permit to better understand the resistance of IL-17-producing cells to AICD and to eventually modulate the inflammatory response caused by the persistence of these cells in autoimmune diseases.

## Materials and Methods

### MS subjects

Patients with relapsing-remitting (RR)-MS in active phase according to the established criteria (Polman and coworkers^41^) were enrolled in the study (*n*=6; four females and three males; aged 38.6±5.3 years; EDSS 2±1.3; disease duration 10.5±3.9 years). Approval by the ethics committee of the Policlinico Tor Vergata, Santa Lucia Foundation and San Camillo Hospital and written informed consent in accordance with the Declaration of Helsinki from all participants were obtained before study initiation. All patients included in the study did not take immunomodulant or immunosuppressive compounds at least 2 months before recruitment. Healthy age-matched and gender-matched controls were included in the study.

### T-helper cell cloning procedure and culture

Peripheral blood samples were purified from buffy coats of healthy adult volunteer blood donors or whole blood of RR-MS patients as previously described.^42^ Single cell sorting of CD4^+^, CD25^+^, CD161^+^ and CCR6^+^ was performed by using a MoFlo high-speed cell sorter (Beckman Coulter, Brea, CA, USA) (Supplementary Figure S1A). Sorted cells were seeded in round-bottom 96-well plates, containing 10^5^ irradiated (9000 rad) allogeneic PBMC as feeder cells, 1 *μ*g/ml PHA and recombinant IL-2 (30 U/ml) for 15 days. Growing cultures were supplemented at weekly intervals with IL-2 (30 U/ml). At day 15, purity of recovered CD4^+^ T-cell clones was verified by staining with anti-CD4 and anti-CD161. Then, pure clones were screened and classified into Th1, Th17, Th1/17, and Th0, on the basis of their ability to produce IFN-*γ*, IL-17, both or neither, respectively (Supplementary Figure S1B).

Selected Th clones of the same Th profile were cultured at a concentration of 1 million/ml, in RPMI containing 5% heat-inactivated FBS and 10% heat-inactivated human serum. Cells were activated through the TCR by using plate bound anti-CD3 (eBioscience Inc., San Diego, CA, USA) 10 *μ*g/ml, soluble anti-CD28 (eBioscience Inc.) 5 *μ*g/ml, anti-CD3/anti-CD28-coated beads (Life Technologies, Paisley, UK) (1 bead/cell), neutralising anti-human FAS (clone ZB4) (Merck Millipore, Darmstadt, Germany) 5 *μ*g/ml, activating anti-human FAS (clone CH11) (Merck Millipore) 0.5 *μ*g/ml, iz-TRAIL (human isoleucine zipper) has been kindly provided by H Walczak^43^ 10 *μ*g/ml and TNF-*α* (eBioscience Inc.) 1 *μ*g/ml in the presence of recombinant IL-2 (Roche, Basel, Switzerland) 30 U/ml and compared with unstimulated cells.

### Flow cytometric analysis

Cell death was quantified by staining for Annexin V (1 *μ*g/ml) and Propidium Iodide (PI) (1 *μ*g/ml) (eBioscience Inc.) and analysed by flow cytometry (FACSCanto; Becton Dickinson, Franklin Lakes, NJ, USA). The combination of Annexin with PI allows discriminating between live cells (Annexin neg- PI neg), early apoptotic (Annexin pos- PI neg) and late apoptotic (Annexin pos- PI pos) cells.

Death receptors expressed by Th clones were analysed by staining with mouse anti-human FAS-FITC (Miltenyi Biotec, Bergisch Gladbach, Germany), mouse anti-human TRAILR (Alexis, Enzo Life Sciences, Farmingdale, NY, USA) and mouse anti-human TNFR1a (Miltenyi Biotec) followed by goat anti-mouse-Alexa 488 (Life Technologies). Samples were acquired on a FACSCanto (Becton Dickinson), and data were analysed with FlowJo (Tree Star, Ashland, OR, USA).

### Immunoblotting

Extracts of TCR-activated Th cells were prepared in immunoprecipitation buffer as previously described.^44^ Protein extracts were then separated by 12% sodium dodecyl sulphate-polyacrylamide gel electrophoresis (SDS-PAGE) and blotted into nitrocellulose membrane (Sigma-Aldrich, St. Louis, MO, USA). Membranes were incubated with the following antibodies overnight at 4 °C: rabbit anti-human polyclonal cleaved caspase-3 (Asp175) (Cell Signaling Technologies Inc., Danvers, MA, USA; 1 : 1000 in 5% non-fat dry milk in phosphate-buffered saline (PBS) pH 7.4), mouse anti-human monoclonal IgG2b caspase-8 (MBL, 1 : 1000 in 5% non-fat dry milk in PBS), mouse anti-human monoclonal IgG1 FASL (Becton Dickinson; 1 : 500 in 5% non-fat dry milk in PBS pH 7.4), mouse anti-human monoclonal IgG1 FLIP (NF6) (Alexis, Enzo Life Sciences: 1 : 500 in 5% non-fat dry milk in PBS pH 7.4); mouse anti-human monoclonal IgG1 Bcl-X (BD Transduction Laboratories; 1 : 500 in 5% non-fat dry milk in PBS pH 7.4); and mouse anti-human IgG1 tubulin (Sigma-Aldrich, 1 : 2000 in 5% non-fat dry milk in PBS pH 7.4). Secondary anti-mouse or anti-rabbit IgGs conjugated to horseradish peroxidase (Amersham, GE Healthcare, Little Chalfont, UK) were incubated with the membranes for 1 h at room temperature at a 1 : 10000 dilution in 5% non-fat dry milk in PBS pH 7.4. Immunostained bands were detected by chemiluminescence (Santa Cruz Biotechnology, Inc., Dallas, TX, USA). Computer-assisted scanning densitometry was used to analyse the intensity of the immunoreactive bands.

### Real-time quantitative RT-PCR

Total RNA was extracted by the RNeasy Microkit (Qiagen, Germantown, MD, USA). A mix containing random hexamers, Oligo dT15m (Promega, Madison, WI, USA) and Super Script II Reverse Transcriptase (Life Technologies) were used for cDNA synthesis. Transcripts were quantified by real-time quantitative PCR on an LC480 (Roche) with Applied Biosystems (Foster City, CA, USA) pre-designed TaqMan Gene Expression Assays and Taqman Gene expression Master Mix (Life Technologies). The following probes were used (identified by Applied Biosystems assay identification number): FASL (HS 00181225_m1), EGR1 (HS 00152928_m1), EGR2 (Hs00166165_m1), EGR3 (Hs00231780_m1), IRF1 (Hs00971960_m1), IRF2 (Hs01082884_m1) and MYC (HS 00153408_m1). For each sample, mRNA abundance was normalised to the amounts of ribosomal protein L-34 (Hs00241560_m1).

### Statistical analysis

All statistical analyses were performed using a two-tailed Student *t*-test using GraphPad Prism software (version5.03, GraphPad Software, La Jolla, CA, USA). Statistical significance was retained for *P-*values <0.05. We used the Pearson correlation coefficient to assess the significance of correlation between mRNA of FASL and selected transcription factors.

## Abbreviations

Th: T-helper
TCR: T-cell receptor
FASL: FAS-ligand
MS: multiple sclerosis
IFN: interferon
IL: interleukin
EAE: experimental autoimmune encephalomyelitis
CNS: central nervous system
AICD: activation-induced cell death
FLIP: caspase-8 (FLICE)-like inhibitory protein
HD: healthy donors

## Notes

DC acted as an Advisory Board member of Merck-Serono, Teva, Bayer Schering, Biogen Idec, Novartis, Almirall and GW Pharmaceuticals and received funding for travelling and honoraria for speaking or consultation fees from Merck-Serono, Teva, Novartis, Bayer Schering, Sanofi-aventis, Biogen Idec and Almirall. He is the principal investigator in clinical trials for Novartis, Merck-Serono, Teva, Bayer Schering, Sanofi-aventis, Biogen Idec and Roche. The remaining authors declare no conflicts of interest.
