# Supplementary material for: FAS-ligand regulates differential activation-induced cell death of human T-helper 1 and 17 cells in healthy donors and multiple sclerosis patients
Source: Cell Death Dis. 2015 May 7;6(5):e1741–. doi: 10.1038/cddis.2015.100 (PMC4669684; doi:10.1038/cddis.2015.100)
Supplement: Supplementary Figure S1 [file cddis2015100x1.ppt]

## Slide 1
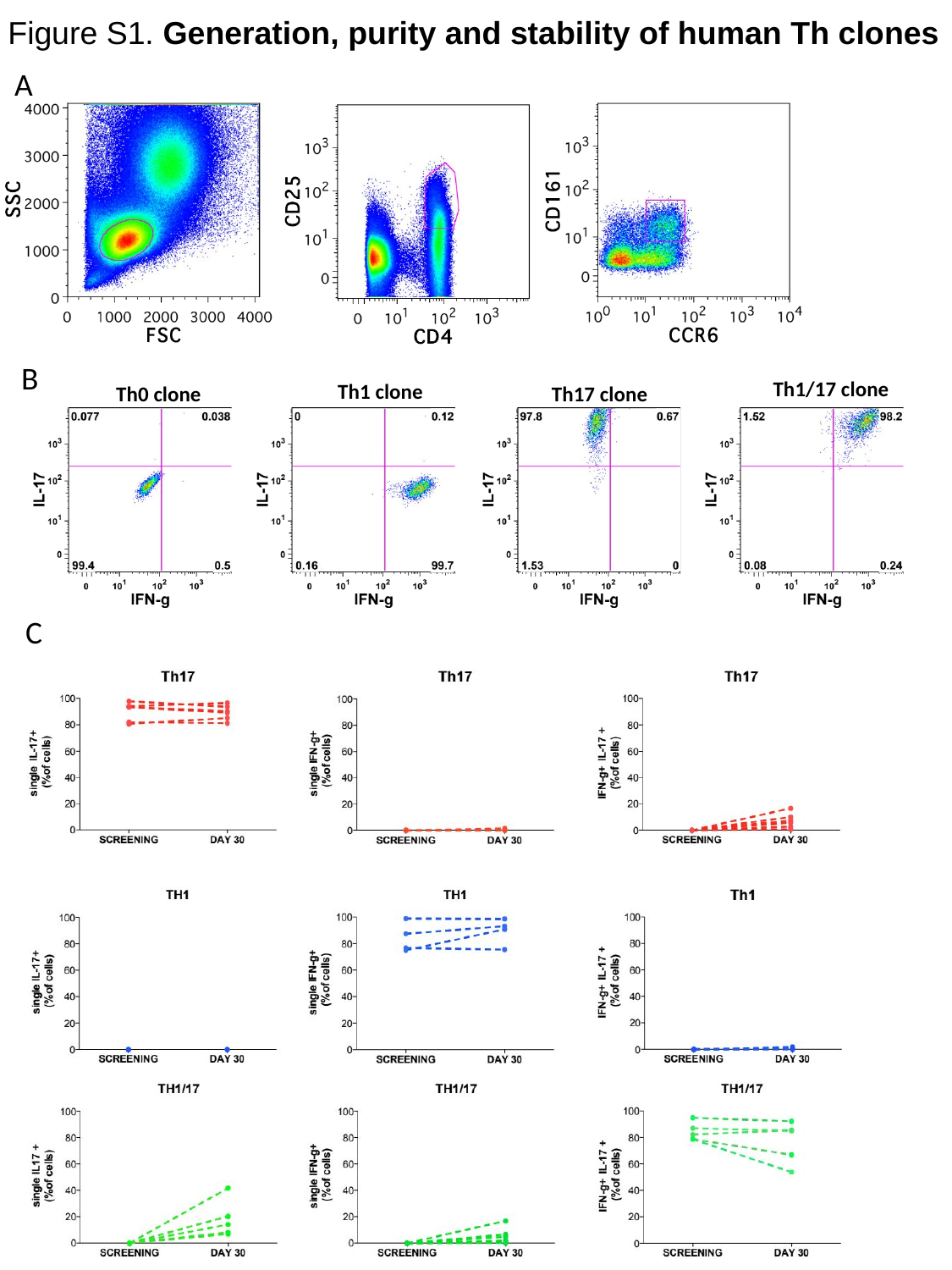

Figure S1. Generation, purity and stability of human Th clones
A
B
Th1/17 clone
Th1 clone
Th17 clone
Th0 clone
C
