# Supplementary figures and images for: FAS-ligand regulates differential activation-induced cell death of human T-helper 1 and 17 cells in healthy donors and multiple sclerosis patients
Source: Cell Death Dis. 2015 May 7;6(5):e1741–. doi: 10.1038/cddis.2015.100 (PMC4669684; doi:10.1038/cddis.2015.100)

## Slide 1
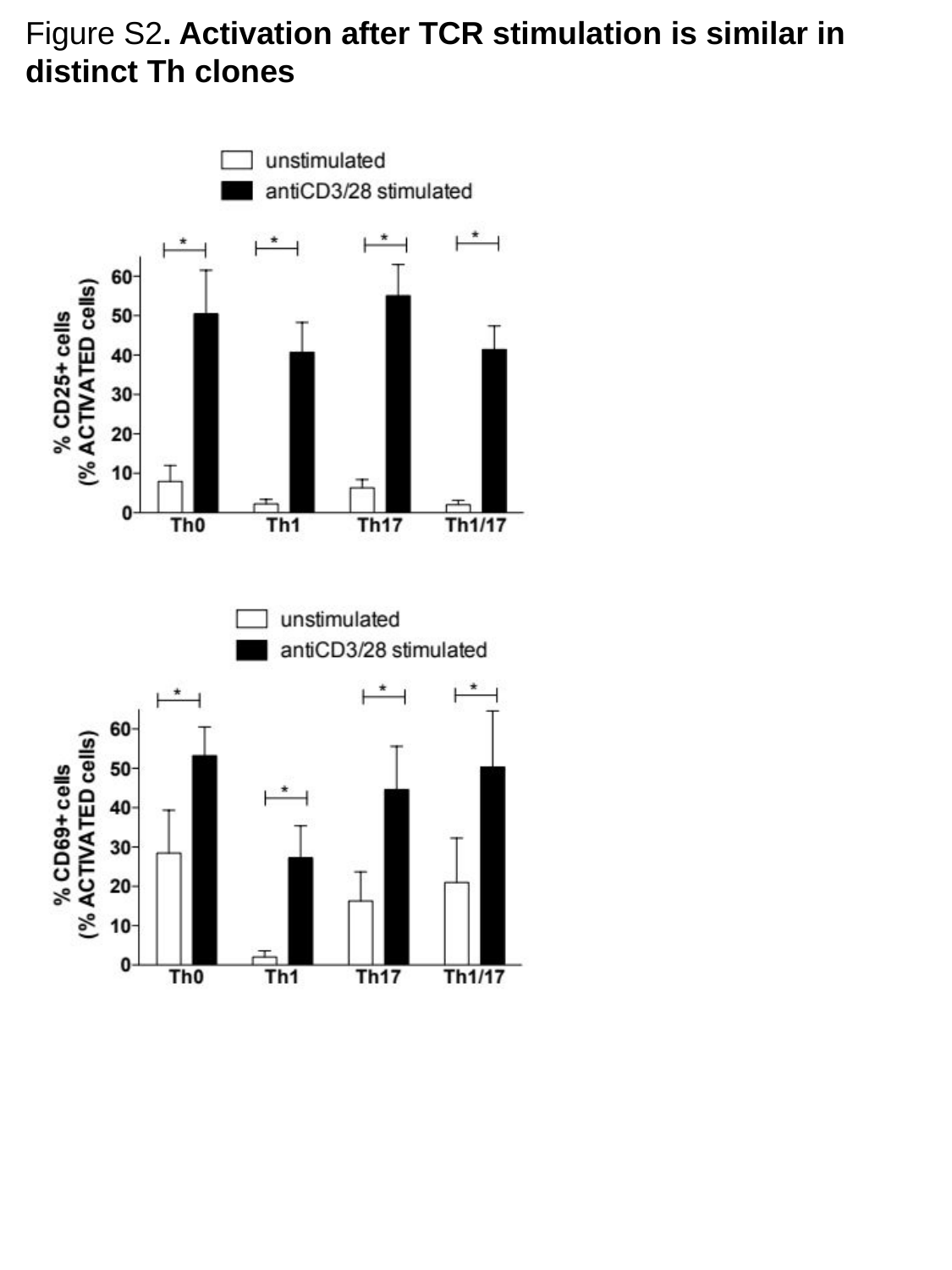

Figure S2. Activation after TCR stimulation is similar in distinct Th clones

Supplement: Supplementary Figure S2 [file cddis2015100x2.ppt]

## Slide 1
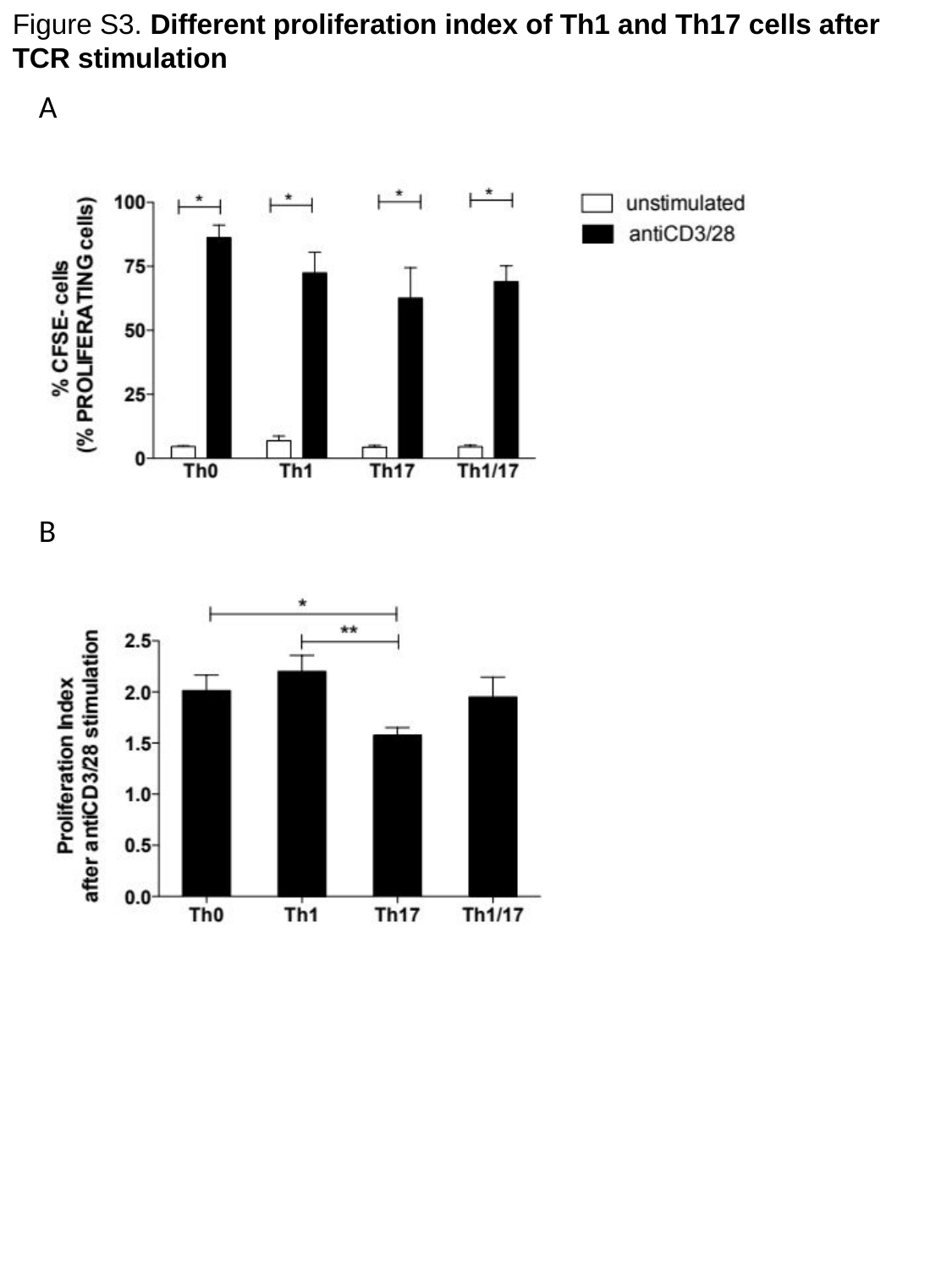

Figure S3. Different proliferation index of Th1 and Th17 cells after TCR stimulation
A
B

Supplement: Supplementary Figure S3 [file cddis2015100x3.ppt]
