# Supplementary material for: FAS-ligand regulates differential activation-induced cell death of human T-helper 1 and 17 cells in healthy donors and multiple sclerosis patients
Source: Cell Death Dis. 2015 May 7;6(5):e1741–. doi: 10.1038/cddis.2015.100 (PMC4669684; doi:10.1038/cddis.2015.100)
Supplement: Supplementary Figure S4 [file cddis2015100x4.ppt]

## Slide 1
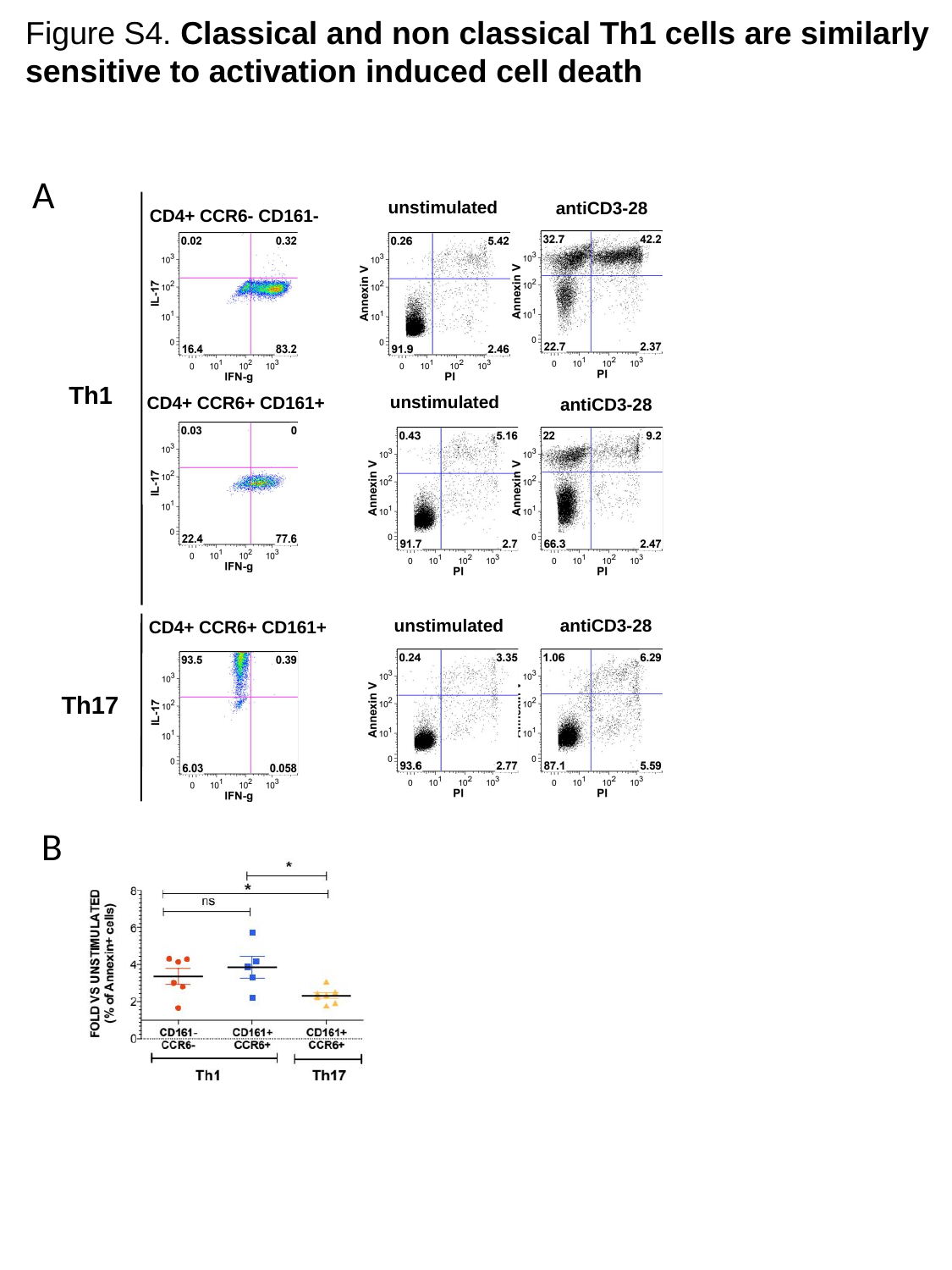

Figure S4. Classical and non classical Th1 cells are similarly sensitive to activation induced cell death
A
unstimulated
antiCD3-28
CD4+ CCR6- CD161-
Th1
unstimulated
CD4+ CCR6+ CD161+
antiCD3-28
unstimulated
antiCD3-28
CD4+ CCR6+ CD161+
Th17
B
