# Supplementary material for: FAS-ligand regulates differential activation-induced cell death of human T-helper 1 and 17 cells in healthy donors and multiple sclerosis patients
Source: Cell Death Dis. 2015 May 7;6(5):e1741–. doi: 10.1038/cddis.2015.100 (PMC4669684; doi:10.1038/cddis.2015.100)
Supplement: Supplementary Figure S5 [file cddis2015100x5.ppt]

## Slide 1
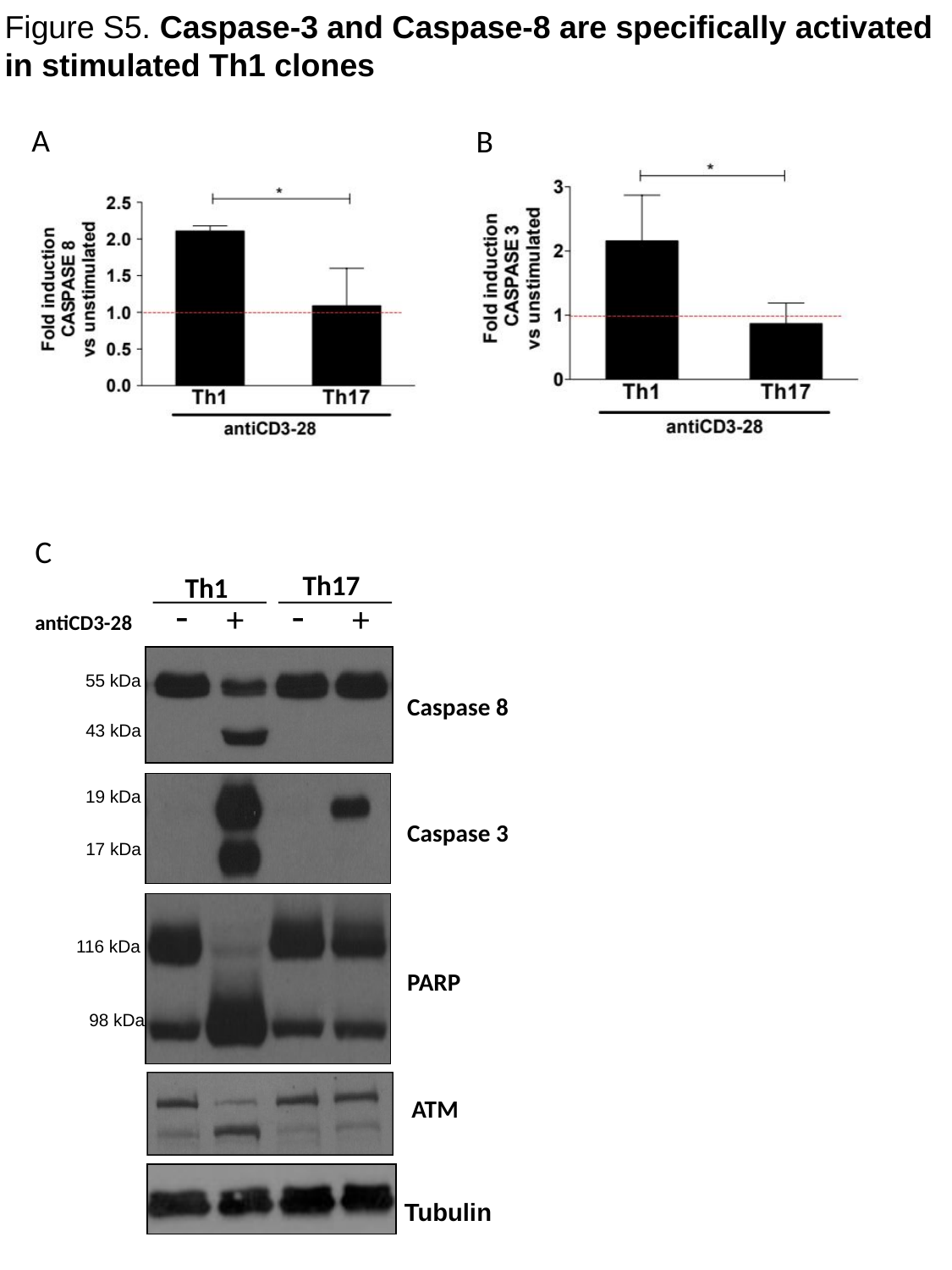

Figure S5. Caspase-3 and Caspase-8 are specifically activated in stimulated Th1 clones
A
B
C
Th17
Th1
- + - +
antiCD3-28
55 kDa
Caspase 8
43 kDa
19 kDa
Caspase 3
17 kDa
116 kDa
PARP
98 kDa
ATM
Tubulin
