# Supplementary material for: FAS-ligand regulates differential activation-induced cell death of human T-helper 1 and 17 cells in healthy donors and multiple sclerosis patients
Source: Cell Death Dis. 2015 May 7;6(5):e1741–. doi: 10.1038/cddis.2015.100 (PMC4669684; doi:10.1038/cddis.2015.100)
Supplement: Supplementary Figure S6 [file cddis2015100x6.ppt]

## Slide 1
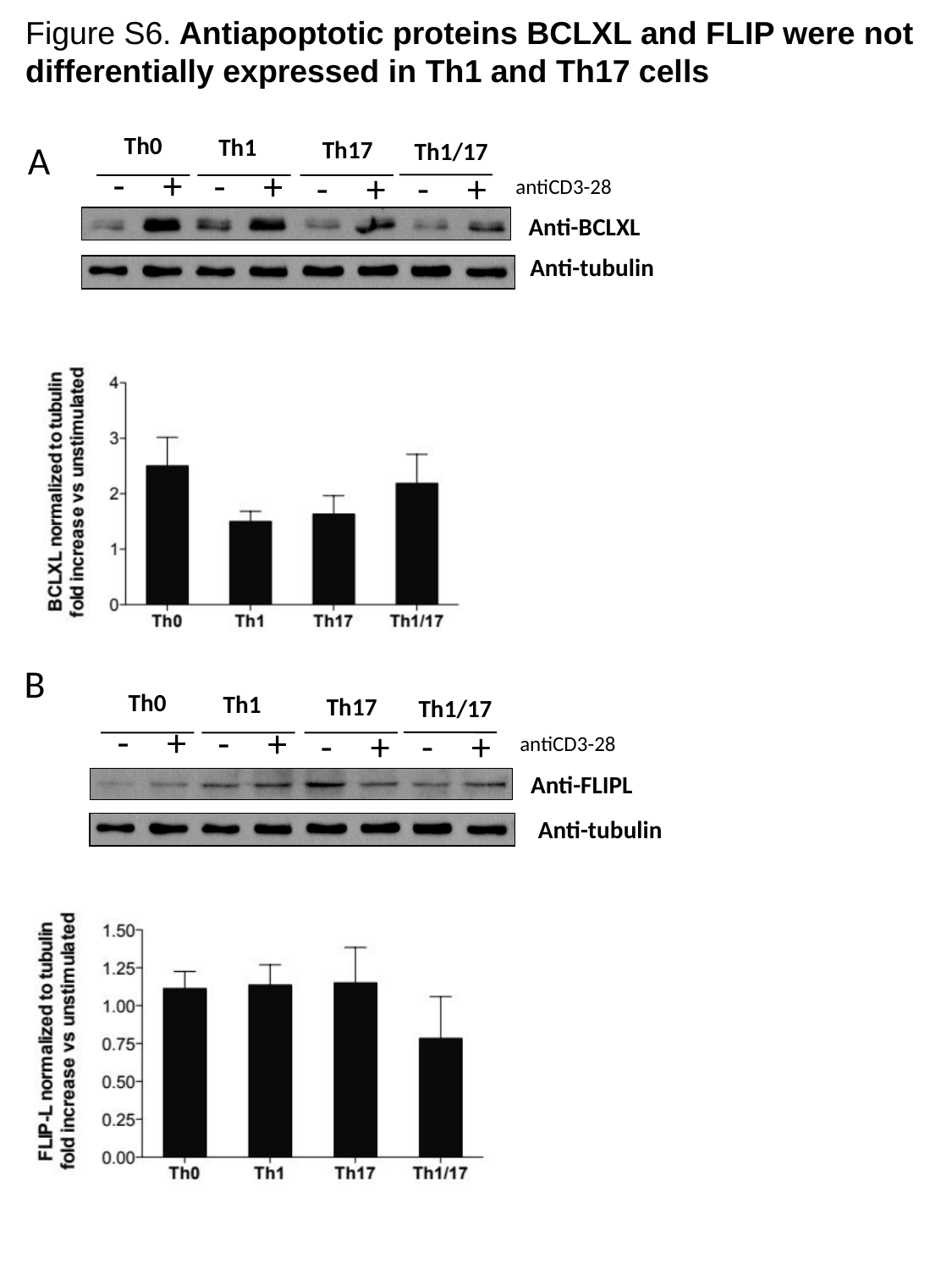

Figure S6. Antiapoptotic proteins BCLXL and FLIP were not differentially expressed in Th1 and Th17 cells
Th0
Th1
Th17
Th1/17
- +
- +
- +
- +
antiCD3-28
A
Anti-BCLXL
Anti-tubulin
B
Th0
Th1
Th17
Th1/17
- +
- +
- +
- +
antiCD3-28
Anti-FLIPL
Anti-tubulin
